# Detection of *P. malariae* using a new rapid isothermal amplification lateral flow assay

**DOI:** 10.1186/s12936-024-04928-9

**Published:** 2024-04-12

**Authors:** Ashenafi Assefa, Kevin Wamae, Christopher M. Hennelly, Billy Ngasala, Meredith Muller, Albert Kalonji, Fernandine Phanzu, Clark H. Cunningham, Jessica T. Lin, Jonathan B. Parr

**Affiliations:** 1 Ethiopian Public Health Institute, Addis Ababa, Ethiopia; 2 Institute of Global Health and Infectious Diseases, University of North Carolina School of Medicine, Chapel Hill, NC USA; 3 Department of Parasitology and Medical Entomology, Muhimbili University of Health and Allied Sciences, Dar es Salaam, Tanzania; 4 SANRU Asbl, Kinshasa, Democratic Republic of the Congo

**Keywords:** RPA, Recombinase polymerase amplification, Lateral flow, Point-of-care testing, Rapid test, Isothermal nucleic acid amplification, *P. malariae*, Diagnostics

## Abstract

**Background:**

While *Plasmodium falciparum* and *Plasmodium vivax* cause the majority of malaria cases and deaths, infection by *Plasmodium malariae* and other *Plasmodium* species also causes morbidity and mortality. Current understanding of these infections is limited in part by existing point-of-care diagnostics that fail to differentiate them and have poor sensitivity for low-density infections. Accurate diagnosis currently requires molecular assays performed in well-resourced laboratories. This report describes the development of a *P. malariae* diagnostic assay that uses rapid, isothermal recombinase polymerase amplification (RPA) and lateral-flow-strip detection.

**Methods:**

Multiple combinations of custom RPA primers and probes were designed using publicly available *P. malariae* genomic sequences, and by modifying published primer sets. Based on manufacturer RPA reaction conditions (TwistDx nfo kit), an isothermal assay was optimized targeting the multicopy *P. malariae* 18S rRNA gene with 39 °C incubation and 30-min run time. RPA product was visualized using lateral strips (FAM-labeled, biotinylated amplicon detected by a sandwich immunoassay, visualized using gold nanoparticles). Analytical sensitivity was evaluated using 18S rRNA plasmid DNA, and clinical sensitivity determined using qPCR-confirmed samples collected from Tanzania, Ethiopia, and the Democratic Republic of the Congo.

**Results:**

Using 18S rRNA plasmid DNA, the assay demonstrates a detection limit of 10 copies/µL (~ 1.7 genome equivalents) and 100% analytical specificity. Testing in field samples showed 95% clinical sensitivity and 88% specificity compared to qPCR. Total assay time was less than 40 min.

**Conclusion:**

Combined with simplified DNA extraction methods, the assay has potential for future field-deployable, point-of-care use to detect *P. malariae* infection, which remains largely undiagnosed but a neglected cause of chronic malaria. The assay provides a rapid, simple readout on a lateral flow strip without the need for expensive laboratory equipment.

**Supplementary Information:**

The online version contains supplementary material available at 10.1186/s12936-024-04928-9.

## Background

Intensive malaria control efforts have yielded progress toward malaria elimination in multiple endemic countries [1]. In some settings, as the burden of *Plasmodium falciparum* and *Plasmodium vivax* malaria declines, less common species such as *Plasmodium malariae* and *Plasmodium ovale* have increased in prevalence as well as public health relevance [2–4]. Malaria elimination requires rapid detection and treatment of all *Plasmodium* species. However, existing rapid methods are not species-specific and have poor sensitivity for these less common species [5]. Though *P. malariae* is known to cause chronic parasitaemia, sometimes lasting years, and causing chronic anaemia and splenomegaly [6], its treatment is straightforward. Thus, its detection and treatment can reduce chronic carriage and contribute to malaria elimination efforts. This study reports a new *P. malariae-*specific diagnostic assay that uses rapid, isothermal recombinase polymerase amplification (RPA) and lateral flow strip detection and has potential for further development into a point-of-care tool.

## Methods

### Primer and probe design

Primers and probes were designed according to manufacturer-suggested best practices [TwistDX(TwistAmpTM nfo), Cambridge, UK]. Primers were designed to meet the following parameters: 30–36 nucleotide (nt) length, 40–50% GC content, 50–100 °C melting temperature, < 5nt mononucleotide repeat length, and 80-500nt amplicon length. Publicly available Pm 18S ribosomal RNA gene sequences from PlasmoDB and NCBI databases were used to design primers using Primer3Plus v3.2. Primers from published literature were also modified and used to guide selection of target regions [7–9]. Probes were designed within the target sequence, with 46-52nt length, 20–80% GC content, and 57–80 °C melting temperature.

### RPA assay protocol

**Fig. 1:**
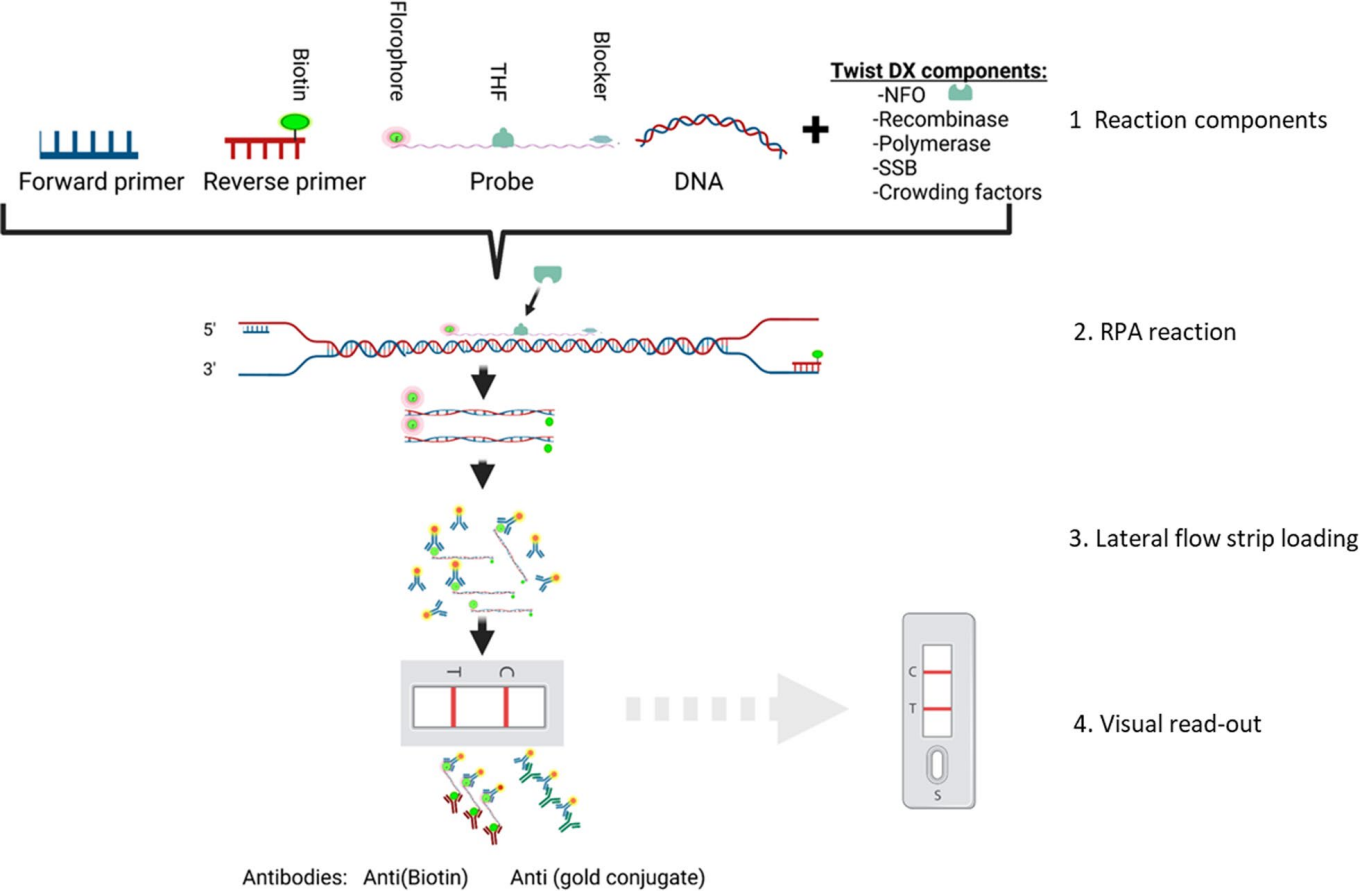
*Plasmodium malariae* lateral-flow based RPA assay schematic. (1) Reaction components include unlabelled forward and 5′-biotinylated reverse primers, 5′-FAM labelled probe with an abasic residue and 3′ blocker, endonuclease IV enzyme (nfo), and template DNA. (2) RPA reaction proceeds via primer-recombinase-SSB complex de-looping, nfo cleavage, polymerase extension that results in FAM- and biotin-labelled double-stranded amplicons. Exponential amplification occurs during 20-min isothermal incubation at 39 °C. (3) Lateral flow strips with a band containing anti-FAM antibodies are loaded with RPA product diluted in buffer containing streptavidin-conjugated gold nanoparticles. (4) Detection of labelled RPA product immobilized on the lateral flow strip is performed by visual inspection using the naked eye. Total reaction time is approximately 35 min. Figure made using *BioRender*

RPA reactions were performed using a reverse primer biotinylated on the 5′ end and an unlabelled forward primer; a 5′-FAM-labelled probe with an abasic residue and 3′ blocker modification. The blocker prevents extension until cleavage of the abasic site by Endonuclease IV (nfo) enzyme. The biotinylated primer and FAM labelled probe form a duplex of double-stranded RPA amplicons that are detectable by sandwich assay, involving a lateral flow strip test band containing anti-FAM antibodies. Direct visualization by naked eye is possible using streptavidin-gold conjugates that bind biotinylated double stranded amplicon captured by anti-FAM antibodies on the lateral flow strip (Fig. 1).

The RPA assay was performed as per manufacturer instructions with slight modifications and using 10 µL reaction volumes (comprising 9 µL of master mix and 1 µL of DNA template). Forty five µL of master mix per five reactions (or proportional equivalent) was prepared, containing 29.5 μL RPA-nfo rehydration buffer, 2.5 μL 280 nM MgOAc, 2.1 μL forward primer (10 μM) and 2.1 μL reverse primer (10 μM), 0.6 μL of probe (10 μM), 1 μL (200 ng/μL) human DNA (for non-clinical samples), and 6.2–7.2 µL molecular-grade water. Reactions were incubated at 39 °C for 30 min with brief intermittent vortexing after 4 min. After amplification, 2 μL of each reaction was immediately diluted with 98 μL of wash buffer in an Eppendorf tube, and a disposable lateral flow strip (Ustar Biotechnologies Ltd, Hangzhou, China REF: D001-03) was dipped into the tube. The result was recorded after 5 min, although results are usually apparent earlier. A test was considered positive when both the test line and control line visualized by the naked eye; and negative only when the control line is visible. When no line appeared, the result was considered invalid. Samples with invalid results were retested.

### Assay optimization

Both TwistDX basic and TwistDX nfo kits were used during optimization of reaction conditions before evaluation of assay performance. TwistDX basic products were denatured using heat (60 °C for 10 min) or purified using commercially available DNA purification kit (Qiagen, Germantown, USA) and visualized by 3% agarose gel electrophoresis. The RPA reaction was performed according to manufacturer’s protocol except with temperature ranging from 34–40 °C for 30 min. For non-clinical samples, 18S rRNA plasmid DNA was combined with 200 ng of human DNA to simulate human sample collection.

### Assessment of assay performance

To determine analytical sensitivity and specificity of the assay, 18S rRNA plasmid DNA of *P. malariae* (MRA-179, Lot: 70031271)*, P. falciparum* (MRA-152-G, Lot: 59201399)*, P. vivax* (MRA-178, Lot: 58067149), and *P. ovale* (MRA-180, Lot: 70043212) obtained from MR4 (BEI Resources, Manassas, USA) was used. To determine limit of detection, *P. malariae* 18S rRNA plasmid DNA was diluted from 10^6^ to 10^–1^ copies/μL. Analytical specificity was evaluated using 18S rRNA DNA from other *Plasmodium* species at high concentration (*P. falciparum* and *P. vivax* at 10^6^ copies/µL, *P. ovale* at 10^3^ copies/µL). Each reaction (RPA and lateral flow detection) was performed with a minimum of four replicates.

Clinical sensitivity and specificity were evaluated using genomic DNA from patients infected with *P. malariae*, *P. falciparum*, *P. ovale* spp., and/or *P. vivax* collected as part of studies in Tanzania, Democratic Republic of Congo (DRC), and Ethiopia [10–12]. Clinical samples were tested using RPA performed in singleton; all sensitivity and specificity estimates were determined using these results. Posthoc, repeat testing was performed for one *P. vivax* sample. For all studies, participants provided informed consent or parental consent was obtained at the time of enrollment. DNA was extracted from dried blood spot samples using Chelex-100, and evaluated for *P. malariae*, *P. falciparum*, *P. ovale* spp., and *P. vivax* infection using real-time PCR targeting the 18S rRNA gene as previously described [10].

## Results

### Selection of primers, probe, and optimized conditions

**Fig. 2:**
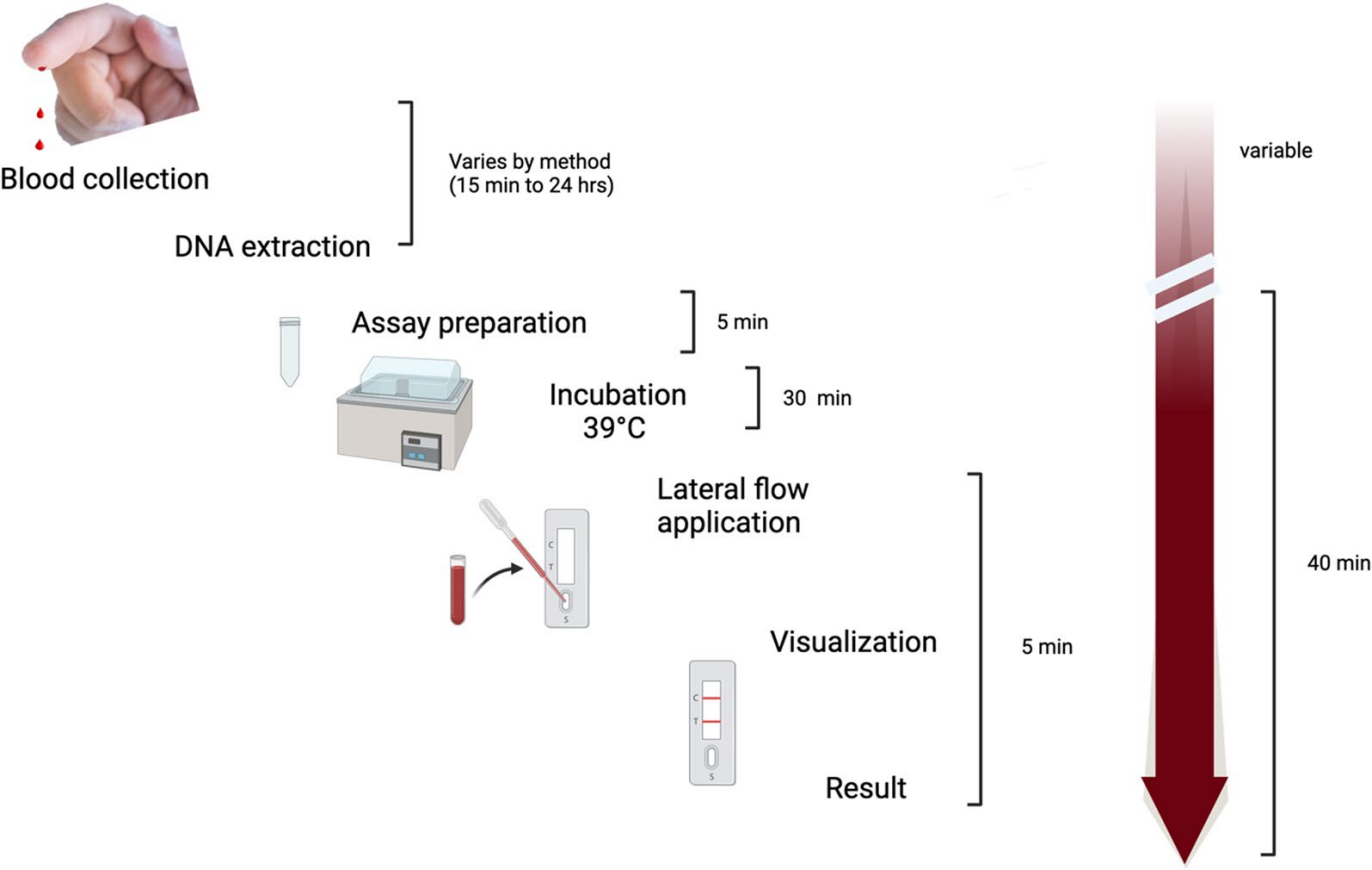
Assay workflow and timeline. Sample collection, DNA extraction (and thawing of DNA and reagents, if necessary) times vary by method. Newer methods enable rapid extraction in ≤ 10 min (e.g., QuickExtract DNA, Biosearch Technologies, Hoddesdon, UK), but at higher cost, whereas conventional Chelex extraction can require up to 24 h at lower cost. Incubation temperature can be varied, though 39 °C was optimal for the assay. Visualization of positive results is often possible in < 2 min but reported at 5 min to standardize assay output. Figure made using *BioRender*

Fifteen primer pairs were evaluated for the current *P. malariae* RPA assay (listed in Additional file 1: Table S1). The length of the primers tested ranged between 30 and 41 nt, with melting temperature ranging from 51 to 62.4 °C and GC content ranging from 20 to 41%. The best performing primers and probe during initial testing using TwistDX basic kits were selected for further evaluation and validation using the TwistDx nfo kit: primers AJMP_7: 5′-ATAACATAGTTGTACGTTAAGAATAACCGC-3′ (forward), AJMP_30: 5′- ATATATAATACTTCGATTAGTTGAGTACCT-3′ (reverse), and probe AJMP_42: 5′- GTTGTACGTTAAGAATAACCGCCAAGGCTTTATTTTTTCTGTTAC-3′. Primer and probe modifications are described in Additional file 2: Table S2. The optimized RPA assay reaction consisted of two-steps (Fig. 2): (1) RPA assay in a heat block at 39 °C for 30 min, and (2) lateral flow assay detection with visualization of results after 5 min (35–45-min total reaction time, not including DNA extraction or reagent preparation).

### Analytical sensitivity and specificity

**Fig. 3:**
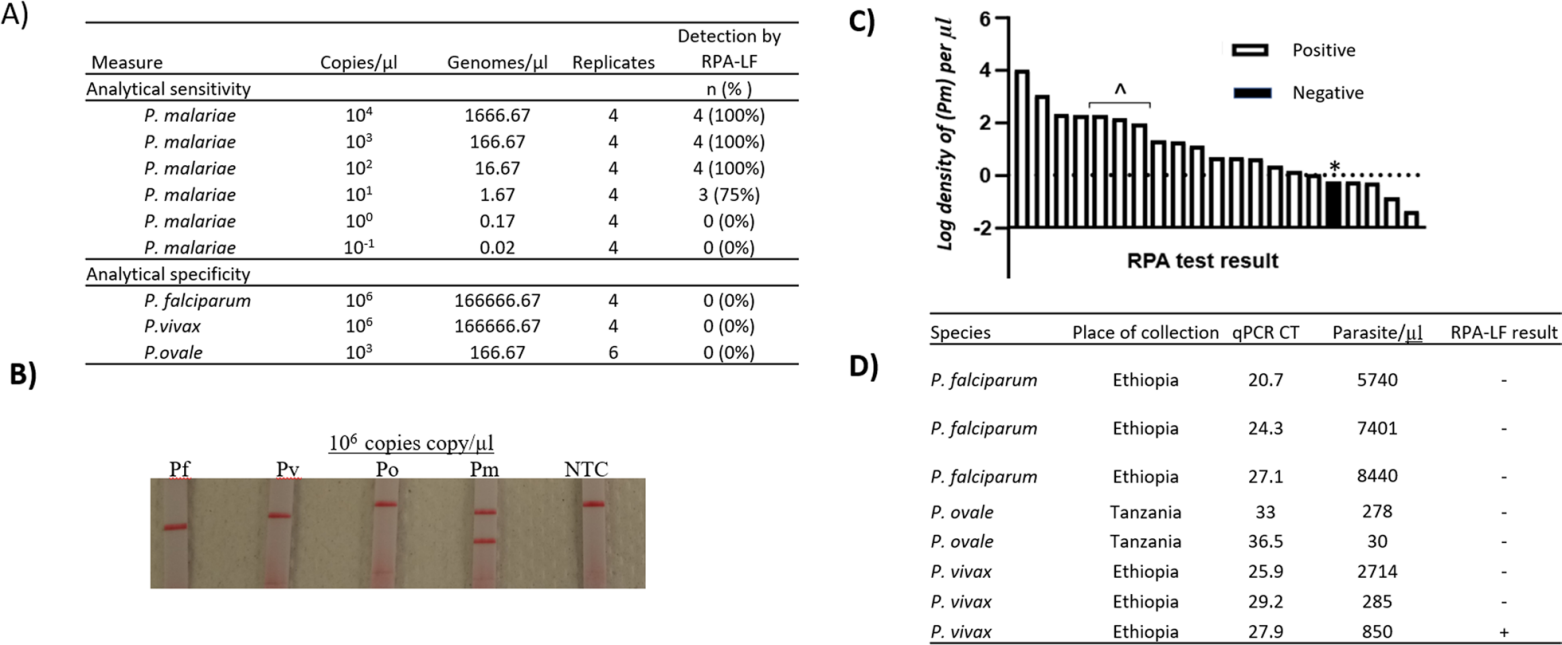
Sensitivity and specificity of the *P. malariae* RPA-lateral flow (RPA-LF) assay. **A** Analytical sensitivity and specificity determined using serially diluted 18S rRNA plasmid copies/µL (equivalent to approximately 1700–0.17 genome equivalents/µL). **B** Example lateral flow read-out by species (Pf, *P. falciparum;* Pv, *P. vivax;* Po, *P. ovale* spp.; Pm, *P. malariae*; NTC, no-template control). **C** Clinical sensitivity determined using 21 Tanzania field samples with qPCR-confirmed *P. malariae* infection with a range of qPCR-determined parasite densities (log_10_). One low-density case was missed (*). Three cases of Pm + Pf co-infection were included (^); the parasite density indicated for these Pm + Pf cases refers only to Pm. **D** Clinical specificity determined using Tanzania and Ethiopia field samples with qPCR-confirmed *P. falciparum*, *P. ovale*, and *P. vivax*

The assay’s analytical sensitivity approached the sensitivity of *P. malariae*-specific PCR assays [8] and achieved perfect analytical specificity (Fig. 3A, B). When tested against serial dilutions of *P. malariae* 18S rRNA plasmid DNA, the lower limit of detection was between 10 and 100 copies/µL (~ 1.7–17 genome equivalents/µL, assuming six copies of 18S rRNA per genome [12, 13], with all replicates containing ≥ 10 *P. malariae* copies/µL detected except for a single replicate. Specificity assessed using high-concentration plasmid DNA for *P. falciparum* (10^6^ genomes/µL), *P. vivax* (10^6^), and *P. ovale* spp. (10^3^) confirmed 100% analytical specificity.

### Clinical sensitivity and specificity

Clinical sensitivity and specificity assessed using field samples from Tanzania and Ethiopia were excellent, with only a single false-negative and false-positive, respectively (Fig. 3C, D). The assay’s clinical sensitivity compared to qPCR was 95.2%, detecting 20 of 21 *P. malariae* qPCR-confirmed field samples (17 mono-infected and three mixed *P. falciparum* and *P. malariae* co-infection) from Tanzania with a range of parasite densities down to 10^0^ and 10^–1^ parasites/µL, corresponding to Ct values ranging 22–41. A single false-negative result was observed in *P. malariae* field sample with parasite density of 0.5 parasites/µL, below the assay’s analytical limits of detection. Clinical specificity was 88% during testing against three *P. falciparum*, two *P. ovale* spp., and three *P. vivax* field samples from Tanzania and Ethiopia. One false-positive result was observed in a *P. vivax* field sample from Ethiopia, with a faint positive band that was not visualized during repeat posthoc testing.

## Discussion

This study describes a new *P. malariae* RPA-lateral flow assay with strong performance when applied to laboratory and field samples, and potential for development for future use in the field. Species-specific point-of-care assays for neglected *Plasmodium* species such as *P. malariae* are not currently available. PCR assays for these species require thermocyclers and materials that are not available in many settings where malaria is endemic. This *P. malariae* RPA-LF assay provides a simple, sensitive, and specific option to detect *P. malariae* in laboratories in low-resource settings.

The *P. malariae*-specific RPA-LF assay achieved limits of detection 10–100 fold better than existing pan-*Plasmodium* lactate dehydrogenase rapid diagnostic tests, with a lower limit of detection of 1.7–16.7 versus 100–1000 parasites/µL, respectively [14]. The assay had perfect analytical specificity and yielded only a single false-negative during testing against field samples. The false-negative result was observed in a sample with low parasite density below the assay’s limit of detection. While the assay detected several other field samples with lower parasite densities, its performance against low-density samples ≤ 10 parasites/µL is expected to be less robust than higher-density samples, but this is also the case with qPCR. Assay performance was comparable to other laboratory-based molecular methods such as PCR and loop-mediated isothermal amplification (LAMP) for *P. falciparum* (Table 1). It was also less complex than CRISPR-based assays that incorporate RPA, though with lower sensitivity for *P. malariae* detection [15, 16]. The assay had excellent specificity, achieving perfect results versus high-concentration plasmid DNA. Its single false-positive during testing of field samples involved a faintly positive test band that was not observed during repeat testing performed as part of post hoc analysis. This suggests that the initial false-positive call was likely a result of contamination during sample processing and RPA-lateral flow testing.

**Table 1:** Comparison of malaria diagnostic methods (adapted from Mbanefo and Kumar 2020)

Method	Microscopy	RDT	PCR	LAMP	RPA (current assay)
Target	N/A	pHRP-2, LDH, Aldolase	18S rRNA	18S rRNA, Mitochondrial DNA	18S rRNA
Sensitivity	95%^a^	85% to 94.8%	98% to 100	98.3% to 100%	95%^a^
Specificity	98%^a^	95.2% to 99%	88% to 94%	94.3% to 100%	88%^a^
Limit of detection	50–200 parasites per μL	0–100 parasites per μL	0.5–5 parasites per μL	1–5 parasites per μL	1.7–16.7 parasites/µL
Cost per test	$0.12–$0.40	$0.85	$7–$8	< $1–$5.3	$2.5–3.5^b,c^
Time	60 min	15–20 min	2 h	30-60 min	35–45 min

Comparisons are against microscopy unless specified. Time estimates do not include sample preparation (see Fig. 2)

^a^Sensitivity and specificity as compared to polymerase chain reaction (PCR)

^b^RPA-LF costs are driven by the TwistAmp nfo kit (approx. $500 per 96 reaction pellets, which can be reconstituted and split for use with five 10 µL reactions each) and lateral flow strips (approx. $2 per unit). Other costs are comparable to PCR, though RPA does not require the up-front equipment costs (thermocycler). Precise estimates per reaction are expected to vary by location

^c^For economical use of resources, we have effectively reduce the reaction volume to 10 µL (comprising 9 µL master mix and a proportional 1 µL of DNA template). Manufacturer recommendations for Twist reagent storage is 4 °C for short periods and − 20 °C for longer periods

RPA provides a rapid method of amplification, reportedly faster than other isothermal amplification methods [17], that can be performed at/near human physiological temperatures. The assay had a total reaction time of less than 40 min and performed best at 39 °C, suggesting opportunities for future use without a heat block (for example, incubation in the axilla or near a simple stove), though these approaches are not yet tested.

Several limitations must be overcome before the current assay is ready for point-of-care use. First, reagent stock outs hampered RPA-lateral flow diagnostic development throughout the COVID-19 pandemic and have not been rectified. Alternatives to TwistDx’s proprietary approaches and kits are now becoming available [18]. Second, the assay requires DNA input and cannot be applied directly to whole-blood samples in its current form. DNA extraction is required prior to performance of the RPA-lateral flow assay and adds time and complexity to sample processing. Advances in simple DNA extraction methods promise rapid turnaround time (< 10 min), but nonetheless add complexity to the workflow. One-pot reactions that eliminate this requirement would simplify assay requirements and improve assay performance. Third, though the assay outperforms pan-*Plasmodium* RDTs and was on par with conventional real-time PCR, it could still miss low-density *P. malariae* infections. This is important because *P. malariae* infections have lower parasite densities than *P. falciparum* and can be missed by commonly used PCR assays. However, it is still likely to pick up the majority of *P. malariae* parasitemia’s detectable by qPCR as well as those contributing to febrile illness [4]. The current assay outperforms pan-*Plasmodium* RDTs, and a future point-of-care version could fill a key gap in the malaria diagnostic portfolio.

Evidence is limited about *P. malariae*, including its true prevalence and distribution, and the extent of its propensity to cause chronic carriage and clinical disease. Simpler diagnostic tools that are closer to the point of care could help resolve some of these questions. They may also contribute to efforts to achieve malaria elimination, as all malaria species need to be detected and addressed. The *P. malariae-*specific RPA-LF assay described here provides a useful laboratory tool for countries seeking to address a key neglected malaria species known for chronic parasitism in low resource settings.

### Supplementary Information

**Additional file 1: Table S1.** List of RPA Primers investigated for P. malariae detection in the study.

**Additional file 2: Table S2.** Primers and probe set and modifications used in the assay: (1) unlabeled forward primer, (2) reverse primer biotinylated on the 5’ end, and (3) 5’-FAM-labelled probe with an abasic residue and 3’ blocker modification.

## Data Availability

Data is provided in Fig. 3 and as Supplementary Material.

## Abbreviations

*PCR*: Polymerase chain reaction
qPCR: Quantitative polymerase chain reaction
*RPA*: Recombinase polymerase amplification
*RPA-LF*: Recombinase polymerase amplification lateral flow
*rRNA*: Ribosomal RNA
*RDT*: Rapid diagnostic test
nt: Nucleotides
NCBI: National Center for Biotechnology Information
LAMP: Loop-mediated isothermal amplification (LAMP)
DRC: Democratic Republic of Congo
CRISPR: Clustered Regularly Interspaced Short Palindromic Repeats

## References

[1] Feachem RGA, Chen I, Akbari O, Bertozzi-Villa A, Bhatt S, Binka F (2019). Malaria eradication within a generation: ambitious, achievable, and necessary. Lancet.

[2] Yman V, Wandell G, Mutemi DD, Miglar A, Asghar M, Hammar U (2019). Persistent transmission of *Plasmodium malariae* and *Plasmodium ovale* species in an area of declining *Plasmodium falciparum* transmission in eastern Tanzania. PLoS Negl Trop Dis.

[3] Sendor R, Banek K, Kashamuka MM, Mvuama N, Bala JA, Nkalani M (2023). Epidemiology of *Plasmodium malariae* and *Plasmodium ovale* spp. in Kinshasa Province, Democratic Republic of Congo. Nat Commun.

[4] Sendor R, Mitchell CL, Chacky F, Mohamed A, Mhamilawa LE, Molteni F (2023). Similar prevalence of *Plasmodium falciparum* and Non-*P. falciparum* malaria infections among schoolchildren, Tanzania. Emerg Infect Dis.

[5] Akala HM, Watson OJ, Mitei KK, Juma DW, Verity R, Ingasia LA (2021). *Plasmodium* interspecies interactions during a period of increasing prevalence of *Plasmodium ovale* in symptomatic individuals seeking treatment: an observational study. Lancet Microbe.

[6] Sutherland CJ, Oguike MC, Smith V, Betson M, Bousema T, Chiodini PL (2010). *Plasmodium ovale* sp. and *Plasmodium malariae* in Africa: difficult items of business on the malaria eradication agenda. Malar J.

[7] Snounou G, Viriyakosol S, Jarra W, Thaithong S, Brown KN (1993). Identification of the four human malaria parasite species in field samples by the polymerase chain reaction and detection of a high prevalence of mixed infections. Mol Biochem Parasitol.

[8] Rutledge GG, Böhme U, Sanders M, Reid AJ, Cotton JA, Maiga-Ascofare O (2017). *Plasmodium malariae* and *P. ovale* genomes provide insights into malaria parasite evolution. Nature.

[9] Goman M, Mons B, Scaife J (1991). The complete sequence of a *Plasmodium malariae* SSUrRNA gene and its comparison to other plasmodial SSUrRNA genes. Mol Biochem Parasitol.

[10] Parr JB, Kieto E, Phanzu F, Mansiangi P, Mwandagalirwa K, Mvuama N (2021). Analysis of false-negative rapid diagnostic tests for symptomatic malaria in the Democratic Republic of the Congo. Sci Rep.

[11] Assefa A, Mohammed H, Anand A, Abera A, Sime H, Minta AA (2022). Therapeutic efficacies of artemether-lumefantrine and dihydroartemisinin-piperaquine for the treatment of uncomplicated *Plasmodium falciparum* and chloroquine and dihydroartemisinin-piperaquine for uncomplicated *Plasmodium vivax* infection in Ethiopia. Malar J.

[12] Markwalter CF, Ngasala B, Mowatt T, Basham C, Park Z, Loya M (2021). Direct comparison of standard and ultrasensitive PCR for the detection of *Plasmodium falciparum* from dried blood spots in Bagamoyo, Tanzania. Am J Trop Med Hyg.

[13] Mercereau-Puijalon O, Barale JC, Bischoff E (2002). Three multigene families in *Plasmodium* parasites: facts and questions. Int J Parasitol.

[14] Hofmann NE, Antunes Moniz C, Holzschuh A, Keitel K, Boillat-Blanco N, Kagoro F (2019). Diagnostic performance of conventional and ultrasensitive rapid diagnostic tests for malaria in febrile outpatients in Tanzania. J Infect Dis.

[15] Cunningham CH, Hennelly CM, Lin JT, Ubalee R, Boyce RM, Mulogo EM (2021). A novel CRISPR-based malaria diagnostic capable of *Plasmodium* detection, species differentiation, and drug-resistance genotyping. EBioMedicine.

[16] Lee RA, Puig HD, Nguyen PQ, Angenent-Mari NM, Donghia NM, McGee JP (2020). Ultrasensitive CRISPR-based diagnostic for field-applicable detection of *Plasmodium* species in symptomatic and asymptomatic malaria. Proc Natl Acad Sci USA.

[17] Lobato IM, O’Sullivan CK (2018). Recombinase polymerase amplification: basics, applications and recent advances. Trends Anal Chem.

[18] Xpedite Diagnostics. Recombinase Aided Amplification Kit for detection using lateral flow strips. 2022. https://www.xpedite-dx.com/

