# Supplementary material for: Detection of P. malariae using a new rapid isothermal amplification lateral flow assay
Source: Malar J. 2024 Apr 12;23:104. doi: 10.1186/s12936-024-04928-9 (PMC11015614; doi:10.1186/s12936-024-04928-9)
Supplement: Supplementary file 1 — Additional file 1: Table S1. List of RPA Primers investigated for P. malariae detection in the study. [file 12936_2024_4928_MOESM1_ESM.docx]

**Table S1**. List of RPA Primers investigated for P. malariae detection in the study

| Name | sequence | Type | Length | Tm (^0^C) | GC (%) | Gene | References |
| --- | --- | --- | --- | --- | --- | --- | --- |
| AJM_01 | TATATGAGTGTTTCTTTTAGATAGCTTCCTT | Left (F) | 31 | 59.9 | 29 | Pm 18S rRNA | Designed /modified |
| AJM_02 | TATGCCGACTAGGTGTTGGATGATAGAGTAAA | Left (F) | 32 | 67.9 | 40.6 | Pm 18S rRNA | Designed /modified |
| AJM_03 | AATCCTACTCTTGTCTTAAACTAGTGAGTTTCC | Right (R) | 33 | 63 | 36.4 | Pm 18S rRNA | Designed /modified |
| AJM_04 | ATATATGAGTGTTTCTTTTAGATAGCTTCCTTC | Left (F) | 33 | 61 | 30.3 | Pm 18S rRNA | Designed /modified |
| AJM_05 | GTTTCTTTTAGATAGCTTCCTTCAGTACCTTAT | Left (F) | 33 | 62.2 | 33.3 | Pm 18S rRNA | Designed /modified |
| AJM_06 | TTCTTTTAGATAGCTTCCTTCAGTACCTTAT | Left (F) | 32 | 61.1 | 31.2 | Pm 18S rRNA | Designed /modified |
| AJM_07 | ATAACATAGTTGTACGTTAAGAATAACCGC | Left (F) | 30 | 56 | 33 | SSUrRNA_Pm_gene (M54897.1) | Snounou et al., 1993 |
| AJM_08 | AAAATTCCCATGCATAAAAAAT TATACAAA | Right(F) | 30 | 51 | 20 | SSUrRNA_Pm_gene (M54897.1) | Designed /modified |
| AJM_09 | AGTAATGCTTTGTATATTTATAACATAGTTG | Left (F) | 31 | 55.8 | 22.6 | SSUrRNA_Pm_gene (M54897.1) | Designed /modified |
| AJM_10 | AACACTCTAATTTACTCAAAGTAACAAAATTC | Right(R) | 32 | 59.5 | 25 | SSUrRNA_Pm_gene (M54897.1) | Designed /modified |
| AJM_11 | CTTATATATGAGTGTTTCTTTTAGATAGCTTCC | Left (F) | 33 | 60.1 | 30.3 | SSUrRNA_Pm_gene (M54897.1) | Designed /modified |
| AJM_12 | CTATTAATCTGTCAATCCTACTCTTGTCTTAAA | Right(R) | 33 | 61.1 | 30.3 | SSUrRNA_Pm_gene (M54897.1) | Rutledge et al., 2017 |
| AJM_13 | CTTATATATGAGTGTTTCTTTTAGATAGCTTCC | Left (F) | 33 | 60.1 | 30.3 | SSUrRNA_Pm_gene (M54897.1) | Goman et al., 1991 |
| AJM_14 | ATTAATCTGTCAATCCTACTCTTGTCTTAAACT | Right(R) | 33 | 61.6 | 30.3 | SSUrRNA_Pm_gene (M54897.1) | Designed /modified |
| AJM_15 | ATATATGAGTGTTTCTTTTAGATAGCTTCCTTC | Left (F) | 33 | 61.3 | 30.3 | SSUrRNA_Pm_gene (M54897.1) | Designed /modified |
| AJM_16 | CTATTAATCTGTCAATCCTACTCTTGTCTTAAA | Right(R) | 33 | 61.1 | 30.3 | SSUrRNA_Pm_gene (M54897.1) | Designed /modified |
| AJM_17 | CTTATATATGAGTGTTTCTTTTAGATAGCTTCC | Left (F) | 33 | 60.1 | 30.3 | SSUrRNA_Pm_gene (M54897.1) | Designed /modified |
| AJM_18 | AGCTATTAATCTGTCAATCCTACTCTTGTCTTA | Right(R) | 33 | 62.4 | 33.3 | SSUrRNA_Pm_gene (M54897.1) | Designed /modified |
| AJM_19 | CTTATATATGAGTGTTTCTTTTAGATAGCTTCC | Left (F) | 33 | 60.1 | 30.3 | SSUrRNA_Pm_gene (M54897.1) | Designed /modified |
| AJM_20 | TTAATCTGTCAATCCTACTCTTGTCTTAAACT | Right(R) | 32 | 61.5 | 31.2 | SSUrRNA_Pm_gene (M54897.1) | Designed /modified |
| AJM_21 | ACATTCTTATATATGAGTGTTTCTTTTAGATAGCTTC | Left (F) | 37 | 58 | 27 | SSUrRNA_Pm_gene (M54897.1) | Designed /modified |
| AJM_22 | GTTACAAATAATTATAAAACTTAACACGTACT | Left (F) | 32 | 56.2 | 21.9 | PmUG01_13030700 | Rutledge et al.,2017 |
| AJM_23 | GTTTAAATCTCTACTTATGCTTAATATATTCTC | Right(R) | 33 | 56.5 | 24.2 | PmUG01_13030701 | Designed /modified |
| AJM_24 | ACTGTTACAAATAATTATAAAACTTAACAC | Left (F) | 30 | 54.1 | 20 | PmUG01_13030702 | Designed /modified |
| AJM_25 | TTTAAATCTCTACTTATGCTTAATATATTCTC | Right(R) | 32 | 55.9 | 21.9 | PmUG01_13030703 | Designed /modified |
| AJM_26 | ACTGTTACAAATAATTATAAAACTTAACAC | Left (F) | 30 | 54.1 | 20 | PmUG01_13030704 | Designed /modified |
| AJM_27 | TTAAATCTCTACTTATGCTTAATATATTCTC | Right(R) | 31 | 54.8 | 22.6 | PmUG01_13030700 | Designed /modified |
| AJM_28 | AATATATAATACTTCGATTAGTTGAGTACCT | Left (F) | 31 | 55.8 | 25.8 | PmUG01_13030700 | Designed /modified |
| AJM_29 | TACTATTTAGAAGAACATGAATAAGAATTT | Right(R) | 30 | 55.5 | 20 | PmUG01_13030700 | Designed /modified |
| AJM_30 | ATATATAATACTTCGATTAGTTGAGTACCT | Right(R) | 30 | 53 | 27 | PmUG01_13030700 | Designed /modified |
| AJM_31 | TCAACACGGGGAAACTCACTAGTTTAAGA | Left (F) | 41 | 59 | 41 | SSUrRNA_Pm_gene (M54897.1) | Designed /modified |
| AJM_40 | ATCACTTGTATTTTTTCTAACTTATTTACAT | Left (F) | 31 | 51 | 19 | Cytochrom (MN175639.1) | Designed /modified |
| AJM_41 | ACATAACCAAATAACAAGTGCTGGAATTGAAG | Right(R) | 32 | 58 | 34 | Cytochrom (MN175639.1) | Designed /modified |
