# Supplementary material for: Detection of P. malariae using a new rapid isothermal amplification lateral flow assay
Source: Malar J. 2024 Apr 12;23:104. doi: 10.1186/s12936-024-04928-9 (PMC11015614; doi:10.1186/s12936-024-04928-9)
Supplement: Supplementary file 2 — Additional file 2: Table S2. Primers and probe set and modifications used in the assay: (1) unlabeled forward primer, (2) reverse primer biotinylated on the 5’ end, and (3) 5’-FAM-labelled probe with an abasic residue and 3’ blocker modification. [file 12936_2024_4928_MOESM2_ESM.docx]

**Table S2**

Primers and probe set and modifications used in the assay: 1) unlabeled forward primer, 2) reverse primer biotinylated on the 5’ end, and 3) 5’-FAM-labelled probe with an abasic residue and 3’ blocker modification.

| Name | Type | Modified sequence |
| --- | --- | --- |
| AJMP_7 | Forward primer | 5’-ATAACATAGTTGTACGTTAAGAATAACCGC-3’ |
| AJMP_30 | Reverse primer | 5’-/5Biosg / ATATATAATACTTCGATTAGTTGAGTACCT-3’ |
| AJMP_42 | Probe | 5’-/FAM-dT/GTTGTACGTTAAGAATAACCGCCAAGGCTT/idSp/TATTTTTTCTGTTAC/3SpC3/-3’ |
